# Proceedings of Cass Co., Ind., Medical Society

**Published:** 1873-07-15

**Authors:** J. A. Adrain, J. H. Goodell

**Affiliations:** President; Logansport, Ind.; Sec’y.; Logansport, Ind.


*Secretary.*

### CASS COUNTY MEDICAL SOCIETY.

LOGANSPORT, Ind., June 25th, 1873.

Many of the physicians of Cass County, Indiana, assembled in the Court House in Logansport, Wednesday, June 25th, pursuant to notice, for the purpose of organizing a County Medical Society.

The meeting was called to order by Dr. Bell, of Logansport, and Dr. Justice called to the chair, with Dr. Goodell, of Walton, as temporary Secretary.

A Committee on Business reported an order of exercises, which was then taken up.

Drs. Coleman, Adrain and Thomas, the Committee on Permanent Organization, reported the following offices to be filled by ballot: President, Vice-President, Secretary, Treasurer, and three Censors.

Drs. Faber, Bell and Washburn, the Committee on Constitution, reported a code, which, after some discussion, was adopted.

The Society next proceeded to elect officers for the coming year as follows: J. A. Adrain, President; Wm. H. Bell, Vice-President; J. H. Goodell, Secretary; J. M. Justice, Treasurer, and Drs. Coleman, Washburn and Thomas, Censors.

Dr. Justice thanked the Society for the honor conferred upon him by electing him temporary Chairman, and introduced Dr. Adrain, the President for the coming year, who made a fine speech on the effect of associated effort, and hoped the examples he had adduced would be incentives to more earnest action in our profession.

The following gentlemen came forward and signed the By-Laws: J. A. Adrain, Armard; Wm. H. Bell, Logansport; J. H. Goodell, Walton; J. M. Justice, Logansport; Jno. Wilds, Logansport; J. B. McElvrey, Curvton; G. W. Nafe, Big Indian; Jno. Herman, Logansport; R. Faber, Logansport; James Thomas, Royal Center; J. V. Hoss, Logansport; J. B. Washburn, Logansport; B. C. Stevens, Logansport.

The Secretary was instructed to have the proceedings published in the Logansport papers, the "Chicago Medical Examiner," and the "Indiana Medical Journal."

The Society then adjourned to meet on the third Wednesday in October, at the Court House in Logansport, at 1 o'clock P.M.

J. A ADRAIN, *President.*

J. H. GOODELL, *Sec'y.*

